# Disability pension and sociodemographic & work-related risk factors among 2.3 million migrants and natives in Finland (2011–2019): a prospective population study

**DOI:** 10.1186/s12889-023-16880-5

**Published:** 2023-10-11

**Authors:** Waseem Haider, Laura Salonen

**Affiliations:** 1 University of Turku, Turku, Finland; 2 Finnish Institute of Occupational Health & University of Turku, Turku, Finland

**Keywords:** Disability pension, Work disability, Migrants, Occupational class, Industrial sector

## Abstract

**Background:**

Increasing employment and immigration have been proposed as possible solutions to tackle the problem of the labour force shortage in aging societies. Ensuring sufficient health and work ability among migrants is a key factor in increasing and maintaining their employment. Many studies have found higher disability pension (DP) rates among migrants compared to natives but such studies lack in determining the risk of DP by occupational class and industrial sector. This study explores the risk of DP and the contribution of sociodemographic and work-related factors between migrants and natives in Finland.

**Methods:**

Full-population panel data obtained from the administrative registers of Statistics Finland were used to study 2.3 million individuals aged 25–60 years in 2010. We calculated hazard ratios (HR) and their 95% confidence intervals (CI) to estimate the risk of having a DP in 2011–2019 using Cox proportional hazard models adjusting for different sociodemographic and work-related factors.

**Results:**

Compared to natives, migrants had a lower risk of a DP (HR 0.58, 95% CI 0.53–0.63). We found great variation between countries of origin, where compared to natives, migrants from refugee-exporting countries (HR 1.37, 95% CI 1.22–1.53) and other non-European countries (HR 1.30; CI 1.18–1.43) had a higher risk of DP, but migrants from other countries did not differ or had a slightly lower risk of DP than natives. The associations between sociodemographic factors and the risk of DP were very similar between natives and migrants.

**Conclusion:**

Migrants had a lower risk of a DP than natives except for migrants from outside Europe. The associations between different sociodemographic and work-related factors and the risk of DP were similar between natives and migrants and did not completely explain the differences in the risk of DP.

**Supplementary Information:**

The online version contains supplementary material available at 10.1186/s12889-023-16880-5.

## Background

Exiting the labour market due to health-related reasons represents a substantial economic burden, averaging 2% of the GDP across OECD countries [1]. Disability pension (DP) is a commonly used indicator of a health-related long-term or permanent exit from the labour market. Premature exit from the workforce due to work disability imposes significant economic burdens in terms of production losses and compensation costs on societies. Simultaneously, it presents challenges to the quality of life for affected individuals. Higher DP rates challenge the sustainability of welfare states by intensifying the burden on an already shrinking workforce, driven by declining fertility rates, an aging population, and a deteriorating dependency ratio [2].

Previous research has identified sociodemographic risk factors for DP including poor health [3], old age [4], low education levels [3, 5, 6], low income [4–6], unemployment [4], and work-related factors such as occupations and industrial sectors characterized by physically demanding labour, unfavorable working conditions, having low job control [5, 6], and working in construction industrial sector [7].

Migrants often work in poorer conditions [8, 9], and are exposed to health hazards more often than natives [8]. They also have a higher risk of experiencing health problems [8–10] and face difficulties in accessing healthcare services [11, 12].

The literature suggests that migrants exit the labour market earlier than natives due to poor health [13], and migrants also face a higher risk of DP compared with natives [14–16]. Among migrants, female sex, old age, low level of education, non-western origin, and a longer length of stay in the host country are associated with a higher risk of DP [14, 15, 17]. However, limited research has explored the relationship between the industrial sector and the risk of DP among migrants, with only a single study focused on individuals aged between 50 and 66 years old [17]. Occupational class significantly relates to DP risk [6], but this remains unexplored in the context of the migrant population. Only two studies [18, 19] used self-reported occupation categories and cross-sectional data to determine the risk of DP among migrants and another study [20] explored the risk of DP in Turkish scaffolders. There is a gap in research investigating the relationship between occupational class, industrial sector, and the risk of DP among migrants using full-population longitudinal data of all working-age migrants.

Unlike other Nordic countries [14, 15] the topic has not been studied in Finland. To our knowledge, this is overall the first study to investigate the risk of DP and occupational class using high-quality full-population longitudinal data, and the first study to investigate the risk of DP and association with sociodemographic factors among migrants in Finland.

To develop well-targeted interventions and preventive measures for tackling work disability, and to enhance migrants’ employability and labour market attachment, it is important to recognize the risk factors and the most vulnerable groups in terms of work disability among migrants. In this study, we investigate the risk of DP among migrants and natives in Finland in 2011–2019, and how the different sociodemographic and work-related factors, migrants’ country of origin, and length of stay in the country contribute to this risk.

## Data and methods

We obtained full-population data from the linked registers of Statistics Finland. Individuals between 25 and 60 years of age who were not on a DP and lived in Finland on 31 December 2010 were followed until they received a DP, an old-age pension, died, or left the country; the follow-up period ended on 31 December 2019. We included individuals aged 25 to 60 to target the working-age population. Individuals under 25 years old are often students and thus less likely to receive DP. In general, receiving DP under the age of 25 is often due to congenital malformations, deformations and chromosomal abnormalities, or severe mental disorders such as schizophrenia, thus making them less comparable to those receiving DP in late adulthood. The upper age limit was kept at 60 years to get at least three follow-up years of the oldest people in the dataset before they became eligible for old-age pensions. We excluded migrants who in 2010 (the baseline year) had been residing in Finland for two or less than two years. To be eligible for social security, an individual needs to have permanent residence status and those in the process of applying for asylum cannot be granted benefits [21, 22]. For migrants to be eligible for DP, a permanent job contract or a fixed-term employment contract of at least two years is considered an indication of permanent residence in Finland [21].

### Disability pension in Finland

In Finland, according to the national pension scheme individuals aged between 16 and 64 years and under an earning-related scheme those at least 17 years old but under their pension age, whose work disability is medically assessed to be long-term or permanent are eligible for a DP. Usually, a DP is granted to those who have obtained a paid sickness allowance of 300 working days. Furthermore, a DP can be full-time (loss of ability to work at least 60%) or part-time (loss of ability to work at least 40%), and fixed-term or permanent [21]. In this study, we were not able to distinguish between the types of DPs.

### Measure of outcome

The primary outcome variable of this study is receiving a DP on the last day of the calendar year. The information on the DP was derived from the basic registers of Statistics Finland. The variable DP was coded as 0 meaning not receiving a DP and 1 meaning receiving a DP.

## Sociodemographic variables

Sex [4, 23], age [4, 23], education level [3, 5, 6, 24], marital status, family structure (25), region [25], and type of residence are all associated with DP. The literature on the risk factors of DP suggests that women are at a higher risk of DP [3, 15]. A higher age [26], not being married [3, 27, 28], or living with a partner have a higher risk of DP [29]. A low level of education is associated with a higher risk of DP [5, 29, 30] but this association is weaker among migrants from low-income countries compared with migrants from Western countries [14]. Österberg & Gustafsson [15] established that family structure, place of origin, place of residence, and length of stay are associated with the risk of DP among migrants.

We divided the variable “age” into three categories: 25–36, 37–48, and 49–60. “Sex” was a binary variable with male and female categories. The variable “education level” was divided into four categories: basic, secondary, lower tertiary, and higher tertiary. The “marital status” variable was divided into four categories: unmarried, married, divorced, and widowed. The “family structure” variable had five categories; single without children, partner with no children, partner with children, single with children, and others (e.g. extended family, blended family, foster family, same-sex partners without child, etc.).

## Work-related variables

Our study incorporated two work-related variables: “occupational class” and “industrial sector”. In general manual workers have a higher risk of DP than non-manual employees [5, 6, 31, 32]. Claussen et al. [19] found a higher risk of DP among migrant manual workers. The industrial sector influences the risk of DP in general and construction trade and extractors jobs have a higher risk of DP compared with managerial positions [7].

There were seven categories in the “occupational class” variable: manual workers, upper non-manual employees, lower non-manual employees, self-employed, students, unemployed, and others. Those with an unknown occupational class were added to the “other” category. The “Industrial sector” was categorized into agriculture, industrial activities (e.g. mining and quarrying, manufacturing, electricity gas, and steam, water supply sewerage, etc.), construction, general services (e.g. wholesale, transportation, accommodation, information & communication, financial & insurance, real state, arts and entertainment, and household as employees), business services (e.g. professional, scientific, technical activities and administrative and support services), public services (e.g. public administration and defence, education, human health & social work), others and out of labour market (e.g. student, conscript, unemployment pensioner, other inactive population). We found a considerable number of missing values in the Industrial sector variable among migrants, notably 44% in the migrant category. In order to address this issue, we examined their principal type of activity in 2010. We classified the missing values in the Industrial sector variable as “out of labour market” for those persons whose primary activity was outside the labour market. Individuals who were employed in their primary type of activity were classified in the “other” industrial sector. This approach enabled us to have a better understanding of migrants’ work status and provide more reliable data for analysis. The “region” variable was classified into Uusimaa (Capital region), South, West, East, and North categories. The variable “type of residence” had two categories urban and rural. During a calendar year, unemployment days were classified as 0 (no unemployment), 1––196, and 197––365. All variables were measured at the end of the baseline year.

## Migrant-specific variables

### Migrant

In this study, “migrant” refers to people who were born abroad, and now live in Finland, or were born in Finland to foreign-born parents. We obtained information on migration status from Statistics Finland’s migration register. We used migrant as a binary variable, with 0 categories for natives and 1 for migrants.

### Region of origin

We followed Jauhiainen & Raivonen’s [33] classification of migrants by country of birth. All European countries, including the United Kingdom, Switzerland, Iceland, and Norway, were placed in the “European countries” category. Russia and the former Soviet Union republics were grouped as “Russia/ex-Soviet Union”, except for Estonia (included in the “European countries” category). All the countries on the Asian continent were amalgamated into the group “Asian countries” except those where one-third of all migrants emigrated for humanitarian reasons (they were added into “refugee-exporting”). Countries from around the globe, where one-third of all migrants emigrated for humanitarian reasons, were placed in the “refugee-exporting” country group. Finally, all of the remaining countries (excluding refugee-exporting countries), including the United States, Canada, and the majority of the African continent, were combined into the “other” category.

### Length of stay

We obtained data on the length of stay of migrants from Statistics Finland’s migration register. We calculated the length of stay in Finland by subtracting the date of entry into Finland from the end of the baseline year. After discounting those with a 0––2 years length of stay, we divided the length of stay into four categories 3––6 years, 7––12 years, 13––18 years, and over 18 years.

### Statistical analysis

The risk of receiving a DP was followed on a yearly level beginning from 2011 and continued until receiving a DP, an old-age pension, or death; the follow-up ended in 2019. We used Chi-square tests to compare the distributions of sociodemographic characteristics and unemployment days between natives and migrants. We investigated the role of migrant-specific factors, such as region of origin and length of stay in Finland, to predict the risk of DP among migrants. To compare the risk of DP among natives and migrants, as well as among migrants, a semiparametric Cox proportional hazard model was used. The model was run for the total population, and separately for natives and migrants. We reported hazard ratios (HR) (exponentiated regression coefficients) with a 95% confidence interval. Cox-Snell residuals were used to assess the model’s fit.

## Results

In the final dataset, 2,321,947 individuals were at risk of a DP in the future. The dataset included 2,216,831 (95%) natives and 105,116 (5%) migrants (Supplementary Table 1). Compared to natives, migrants were on average younger, had a lower level of education, were more often manual workers, unemployed, or in “other” occupational class, were outside the labour market, lived in Uusimaa, and had more unemployment days. Around 43% of the migrants came from Russia/ex-Soviet Union countries and EU countries, and around 60% had been in Finland for less than 13 years.

Between 2011 and 2019, around 5.8% of the study population had a DP during the follow-up, i.e. 6.0% (N = 134,057) of natives and 3.8% (N = 3986) of migrants (Supplementary Table 1). Among those who had DP during the follow-up, migrants were on average younger than natives, had a lower level of education, were unemployed or “other” by their main economic activity status, were out of the labour market, lived more often in Uusimaa, and had more unemployment days than natives who had DPs during the follow-up. 42% of migrant DP recipients were from Russia/ex-Soviet Union, and around 53% had lived in Finland for more than 13 years.

**Table 1:** Fully-adjusted hazard ratios (HR) and 95% confidence intervals (CI) for the risk of disability pension (DP) in the follow-up period 2011–2019

Variables	Migrants			Natives			Total population		
	HR	CI (95%)	*Sig.* ^*1*^	HR	CI (95%)	*Sig.*	HR	CI (95%)	*Sig.*
*Migrant status*									
Natives (ref.)									
Migrants							0.58	0.53–0.63	***
*Sex*									
Male (ref.)									
Female (ref.)	1.17	1.09–1.26	***	1.13	1.12–1.15	***	1.14	1.12–1.15	***
*Age category*									
25–34 years (ref.)									
35–44 years	2.58	2.33–2.87	***	2.20	2.16–2.24	***	2.22	2.17–2.26	***
45–54 years	5.53	4.96–6.17	***	4.47	4.39–4.55	***	4.51	4.43–4.59	***
*Educational level*									
Basic (ref.)									
Secondary	1.05	0.97–1.13		0.88	0.86–0.88	***	0.89	0.88–0.90	***
Lower tertiary	0.87	0.78–0.97	*	0.56	0.55–0.57	***	0.57	0.55–0.59	***
Higher tertiary	0.61	0.53–0.70	***	0.41	0.40–0.42	***	0.42	0.41–0.43	***
*Marital Status*									
Unmarried (ref.)									
Married	1.01	0.87–1.22		1.14	1.12–1.16	***	1.13	1.11–1.15	***
Divorced	1.17	1.03–1.32	*	1.22	1.20–1.25	***	1.23	1.20–1.25	***
Widowed	0.94	0.74–1.21		0.88	0.85–0.93	***	0.88	0.85–0.93	***
*Family structure*									
Single, no children (ref.)									
Partner, no children	0.81	0.66–0.91	**	0.84	0.83–0.86	***	0.83	0.83–0.86	***
Partner with children	0.72	0.60–0.81	***	0.67	0.66–0.69	***	0.67	0.66–0.69	***
Single with children	0.76	0.65–0.92	***	0.87	0.84–0.89	***	0.84	0.82–0.87	***
Other	0.77	0.57–0.82	***	0.95	0.93–0.97	***	0.91	0.91–0.96	***
*Occupational class*									
Manual workers (ref.)									
Upper non-manual employees	0.43	0.36–0.51	***	0.50	0.48–0.51	***	0.49	0.48–0.51	***
Lower non-manual employees	0.72	0.66–0.89	***	0.72	0.70–0.73	***	0.72	0.70–0.73	***
Self-employed	0.76	0.64–0.97	***	0.72	0.70–0.74	***	0.72	0.70–0.74	***
Students	0.71	0.47–0.78	**	0.59	0.57–0.62	***	0.59	0.57–0.62	***
Unemployed	0.99	0.84–1.16		0.58	0.58–0.62	***	0.59	0.57–0.61	***
Other	1.38	1.17–1.61	***	1.15	1.11–1.18	***	1.15	1.12–1.19	***
*Industrial sector*									
Agriculture (ref.)									
Industrial activities	0.70	0.53–0.94	*	0.78	0.75–0.81	***	0.78	0.75–0.81	***
Construction	0.69	0.49–0.97	*	0.95	0.88–0.97	*	0.95	0.91–0.98	*
General Services	0.72	0.54–0.96	*	0.89	0.86–0.91	***	0.88	0.86–0.91	***
Business services	0.87	0.66–1.15		0.97	0.87–1.01		0.97	0.93–1.01	
Public services	0.87	0.66–1.15		1.33	1.28–1.37	***	1.33	1.28–1.37	***
Other	0.58	0.43–0.78	***	1.18	1.12–1.21	***	1.17	1.12–1.21	***
Out of labour market	0.92	0.76–1.28		2.13	2.04–2.22	***	2.07	1.99–2.15	***
*Region of residence*									
Uusimaa (ref.)									
South	0.97	0.81–1.09		1.14	1.12–1.17	***	1.13	1.12–1.17	***
West	1.06	0.94–1.16		1.17	1.14–1.18	***	1.16	1.15–1.19	***
East	1.04	0.83–1.22		1.33	1.30–1.36	***	1.32	1.30–1.36	***
North	1.30	1.12–1.51	**	1.42	1.39–1.45	***	1.40	1.39–1.45	***
*Type of region*									
Urban (ref.)									
Rural	1.10	0.98–1.22		1.04	1.03–1.06	***	1.05	1.03–1.06	***
*Unemployment days*									
0 (ref.)									
1-196	1.10	0.99–1.19		1.32	1.30–1.35	***	1.32	1.20–1.34	***
Over 196	1.17	1.03–1.32	*	1.15	1.12–1.18	***	1.16	1.13–1.19	***
*Country of origin*							(ref. Natives)		
European countries	(ref. European countries)						1.01	0.94–1.07	
Russia /ex-Soviet Union	1.00	0.89–1.13					0.87	0.78–0.96	**
Asian countries	1.07	0.90–1.19					0.87	0.77–0.98	*
Refugee-exporting countries	1.81	1.57–2.09	***				1.37	1.22–1.53	***
Other	1.69	1.46–1.94	***				1.30	1.18–1.43	***
*Length of stay (years)*									
3–6 (ref.)									
7–12	1.02	0.93–1.13							
13–18	1.18	1.07–1.31	**						
Over 18	1.29	1.15–1.43	***						

^1^ p−value * <0.05, ** <0.01, *** <0.001

After mutually adjusting for all the covariates migrants had a lower risk of a DP with an HR of 0.58 (95% CI 0.53–0.63) than natives (Table 1). In general, female sex, old age, low education level, being widowed, living alone, having “other” as an occupational class, being out of labour market and living in Northern Finland and rural areas, and having an unemployment history were associated with a higher risk of a DP. Most sociodemographic factors had a similar association with a DP among natives and migrants.

The main differences between migrants and natives in their risk of a DP were related to educational level, marital status, occupational class, industrial sector, living area, and unemployment days. In migrants, secondary level educated did not differ in their risk of DP from those with basic education, as among natives they had a lower risk of DP. Unlike natives, widowed and married did not differ in the risk of DP from unmarried migrants. Compared to natives, migrants showed smaller variations in the risk of DP across different occupational classes. Among migrants, self-employed had a lower risk of DP followed by students and upper non-manual employees. Both in natives and migrants the “other” category of occupational class was associated with the highest risk of DP but the risk was higher among migrants. In migrants, the differences in the risk of a DP between different industrial sectors were relatively small due to large confidence intervals. In migrants, working in “other” industries was associated with a lower risk of DP, but in natives with a higher risk of DP than working in Agriculture. Further, compared to agriculture workers, migrants who were out of the labour market did not differ in their risk of DP whereas natives who were out of the labour market had the highest risk of DP. Living in rural areas was associated with a higher risk of DP than living in urban areas, but only among natives. Only very long-term unemployment was associated with a higher risk of DP among migrants, but in natives, any unemployment increased the risk of DP (Table 1).

Migrants from European countries did not differ in their risk of DP from natives, while those from Russia/ex-Soviet Union countries (HR 0.87; 0.78–0.96) and Asian countries (HR 0.87; 0.77–0.98) had a slightly lower risk of DP. Migrants from refugee-exporting countries had a higher risk of a DP 1.37 (HR 1.37 95: CI 1.22–1.53) compared to natives, as did those who were from other countries (HR 1.30; CI 1.18–1.43) (Table 1).

Among migrants, compared to those from European countries, a higher risk of DP was found among those from refugee-exporting countries (HR 1.81, 95% CI 1.57–2.09) and from “other” countries (HR 1.69; CI 1.46–1.94). Living in Finland for longer than 12 years increased the risk of DP (Table 1).

## Discussion

We analyzed the risk of DP among both natives and migrants and studied how different sociodemographic, work-related, and migrant-specific factors were associated with DP in Finland from 2011 to 2019. We found a considerably lower risk of DP among migrants compared to natives, except for migrants from refugee-exporting countries or other non-European countries, even after adjusting for multiple sociodemographic and work-related variables. We further found that different sociodemographic variables were very similarly associated with the risk of DP among natives and migrants, with only a few exceptions.

While no previous studies on the risk of DP among migrants have been conducted in Finland, a recent Finnish study found similar results, showing that migrants working in the health sector had fewer sickness absences than their Finnish-born colleagues [34]. The overall lower risk of DP among migrants could be related to the healthy migrant effect (migrants, especially those migrating due to work and studies are generally healthier than the native population), migrants’ unfamiliarity with the social security and benefits system, fear of stigma or loss of income that can lower their likelihood of applying for a DP, even though migrants have a higher risk of ill health [9, 35, 36] and lower use of health care services [12, 37].

However, we found important differences in the risk of DP between migrants from different countries. Migrants from other European countries did not differ from natives in their risk of DP, while migrants from Russia or ex-Soviet Union countries and Asian countries had a slightly lower risk of DP. These results were contradictory to previous findings [14] as they found a higher risk of DP among Asian migrants compared with natives. In this study, most migrants were from Russia and ex-Soviet Union countries, which probably largely explains the overall lower risk of DP among migrants compared to natives. They have typically lived in Finland for a long period and compared to other migrants, are more likely to have higher education, pre-existing family ties, and other factors that facilitate their (cultural) adaptation. In other words, the composition of the migrant population in terms of their country of origin, the reason for immigration, labour market attachment, industry, working conditions, and health can differ between Finland and other Nordic countries [38]. It is also likely that disparities in welfare systems or integration processes exist between Finland and the other Nordic nations, which could explain the country differences in the findings.

We also found that migrants from refugee-exporting countries and other non-European countries had a higher risk of DP than natives or other migrants. These results were partially contradictory to previous findings of the studies conducted on the risk of DP [14, 15]. Österberg & Gustafsson [15] found a higher risk of DP among migrants from European countries (e.g. Greece, Former Yugoslavia, Finland, Denmark, Poland, and Hungary) in Sweden whereas in our study we found that migrants from refugee-exporting countries (which did not include these countries) had the higher risk of DP. Claussen et al. found the highest risk of DP among migrants from the Middle East and North Africa compared with natives in Norway. This was partially in line with the results of our study as some of the Middle Eastern and North African countries were included in the refugee exporting category that had the highest risk of DP. The higher risk of DP among migrants from refugee-exporting countries could be due to the humanitarian reasons for migration. They might have experienced traumatic events like war, persecution, or displacement that can lead to physical or psychological impairments. These experiences can hamper maintaining their ability to work [39] therefore increasing the risk of seeking a DP. It is possible that many of the migrants from “other” countries, all from outside Europe, have moved to Finland due to humanitarian reasons.

In addition to the country of origin, we also find that the risk of DP increased with the length of stay, which is in line with previous studies [40]. Among those who had DP most had a relatively long length of stay in Finland, compared to the total migrant population. This likely reflects the integration process and better attachment to the labour market, which in turn, provides access to occupational health care, social support and awareness of the benefits system, and financial resources. It is also associated with aging (older people suffer more health problems), and the accumulation of work-related injuries and illnesses over time.

While most sociodemographic factors had a similar association with DP in both migrants and natives, we also found some differences in work-related factors. We found that migrants had a lower risk of DP in all occupational classes except “other” occupations compared with natives. We also found that working in the public services, “other” industry, or being out of labour market was associated with a higher risk of DP than working in agriculture among the natives but not among the migrants. A possible explanation could be the considerably lower number of migrants in the public service industry and the fact that the data on the industrial sector of more than 40% of migrants were missing. Natives who are out of labour market might differ from migrants who are out of labour market in terms of occupational history, labour market attachment, health, health literacy, workability, and in their access to health care services.

### Strengths and limitations

The use of relatively recent data from a full population register with a long follow-up, little or no missing values and attrition were the strengths of this study. A DP that is based on objective measurements rather than self-reported measurements reduces bias. Furthermore, including the category of the refugee-exporting country provided valuable and novel information on the relationships between the country of origin and a DP. The study’s limitations included a lack of information on health and health behavior, both of which are important predictors of a DP. We also lacked information on the migrants’ residency status, which could have explained some of the risk of DP. Administrative registers also lack information on awareness of the benefits system. Finally, due to the study’s observational nature, no causal interpretations can be drawn from the findings. More research is needed to provide explanations for these findings. More research is also needed on migrants’ health, healthcare utilization, and use of other social security benefits, such as sick leave. Understanding migrants’ health and work (dis)ability, as well as their need for assistance, is critical to maintaining their quality of life and increasing their labour market participation. In addition to large-scale quantitative research, qualitative research could provide insights into the lived experiences and perspectives of migrants, which would contribute to an increased risk of DP.

## Conclusion

We investigated the risk of DP and how different sociodemographic and work-related factors were associated with DP among natives and migrants between the years 2011 and 2019. Migrants had a lower risk of DP than natives, which is only partially in line with previous findings. This risk varied largely between migrants from different regions of origin and their length of stay in the country. In general, different sociodemographic factors were similarly associated with the risk of DP among migrants and natives, but we also found some differences. However, even accounting for many different sociodemographic factors and work-related factors, the differences in the risk of DP between natives and migrants remained significant. More research is needed to provide causal explanations for the findings. Furthermore, the findings of the study could inform the development of culturally sensitive and inclusive policies and programs to reduce the risk of DP among migrants, particularly those from refugee-exporting countries and outside Europe.

### Electronic supplementary material

Below is the link to the electronic supplementary material.

Supplementary Material 1

## Data Availability

The data of this study are not available from the corresponding author due to data protection laws and regulations. Data are available from the register data (Statistics Finland) holders upon reasonable request. For the availability of data and materials, Statistics Finland can be contacted at tutkijapalvelut@stat.fi.

## Abbreviations

CI: Confidence Interval
DP: Disability pension
EU: European Union
HR: Hazard Ratio
Kela: The Social Insurance Institution of Finland
