# Supplementary material for: Disability pension and sociodemographic & work-related risk factors among 2.3 million migrants and natives in Finland (2011–2019): a prospective population study
Source: BMC Public Health. 2023 Oct 11;23:1977. doi: 10.1186/s12889-023-16880-5 (PMC10568789; doi:10.1186/s12889-023-16880-5)
Supplement: Supplementary file 1 — Supplementary Material 1 [file 12889_2023_16880_MOESM1_ESM.docx]

**Supplementary Table 1** The distribution of sociodemographic and work-related factors, and migrant-specific characteristics in 2010 and disability pension (DP) from 2011-2019

|  | Migrants | | | | Natives | | | |
| --- | --- | --- | --- | --- | --- | --- | --- | --- |
|  | Total | | DP | | Total | | DP | |
|  | N | *%* | N | *%* | N | *%* | N | *%* |
| Total | 105,116 | 100 | 3986 | 100 | 2,216,831 | 100 | 134,057 | 100 |
| *Sex* | | | | | | | | |
| Men | 52,417 | 50 | 2100 | 46 | 1,114,863 | 50 | 63,175 | 47 |
| Women | 52,669 | 50 | 2514 | 54 | 1,101,968 | 50 | 70,882 | 53 |
| *Age category* | | | | | | | | |
| 25-36 years | 41,585 | 40 | 581 | 15 | 731,302 | 33 | 17,189 | 13 |
| 37-48 years | 39,782 | 38 | 1495 | 37 | 742,224 | 33 | 35,065 | 26 |
| 49-60 years | 23,749 | 22 | 1910 | 48 | 743,305 | 34 | 81,803 | 61 |
| *Educational level* | | | | | | | | |
| Basic | 47,080 | 45 | 1809 | 45 | 302,019 | 13 | 30,714 | 23 |
| Secondary | 29,577 | 28 | 1359 | 34 | 1,012,674 | 46 | 73,163 | 54 |
| Lower Tertiary | 15,331 | 15 | 556 | 14 | 611,675 | 28 | 23,965 | 18 |
| Higher Tertiary | 13,128 | 12 | 262 | 7 | 290,463 | 13 | 6215 | 5 |
| *Marital Status* | | | | | | | | |
| Unmarried | 22,529 | 21 | 527 | 13 | 777,993 | 35 | 37,448 | 28 |
| Married | 62,855 | 60 | 2347 | 59 | 1,144,421 | 52 | 66,947 | 50 |
| Divorced | 18,455 | 18 | 1017 | 26 | 273,059 | 12 | 27,440 | 20 |
| Widowed | 1277 | 1 | 95 | 2 | 21,358 | 1 | 2222 | 2 |
| *Family Structure* | | | | | | | | |
| Single, no children | 14,364 | 14 | 839 | 21 | 420,593 | 19 | 33282 | 25 |
| Partner, no children | 17,657 | 17 | 769 | 19 | 555,965 | 25 | 40935 | 31 |
| Partner with children | 44,380 | 42 | 1446 | 36 | 958,517 | 43 | 40864 | 30 |
| Single with children | 8437 | 8 | 412 | 11 | 126,313 | 6 | 9028 | 7 |
| Other | 20,278 | 19 | 520 | 13 | 155,413 | 7 | 9948 | 7 |
| *Occupational class* | | | | | | | | |
| Manual workers | 28,955 | 28 | 1191 | 30 | 531,204 | 24 | 40,327 | 30 |
| Upper non-manual employees | 13,401 | 13 | 205 | 5 | 437,513 | 20 | 11,061 | 8 |
| Lower non-manual employees | 17,287 | 16 | 524 | 13 | 718,063 | 32 | 38,414 | 29 |
| Self-employed | 8776 | 8 | 260 | 7 | 208,234 | 9 | 12,035 | 9 |
| Students | 6779 | 6 | 199 | 5 | 67,381 | 3 | 3679 | 3 |
| Unemployed | 17,759 | 17 | 1121 | 28 | 197,096 | 9 | 20,731 | 15 |
| Other | 12,159 | 12 | 486 | 12 | 57,340 | 3 | 7810 | 6 |
| *Industrial sector* | | | | | | | | |
| Agriculture | 1233 | 1 | 68 | 2 | 68,019 | 3 | 4669 | 4 |
| Industrial activities | 8965 | 8 | 283 | 7 | 311,950 | 14 | 14,850 | 11 |
| Construction | 3313 | 3 | 96 | 3 | 120,186 | 6 | 7318 | 5 |
| General Services | 21,727 | 21 | 648 | 16 | 536,956 | 24 | 23,833 | 18 |
| Business services | 10,121 | 10 | 335 | 8 | 199,038 | 9 | 9538 | 7 |
| Public services | 13,427 | 13 | 492 | 12 | 556,168 | 25 | 34,085 | 25 |
| Other | 9867 | 9 | 329 | 8 | 157,738 | 7 | 10,469 | 8 |
| Out of labour market | 36,463 | 35 | 1735 | 44 | 226,776 | 12 | 29,295 | 22 |
| *Region of residence* | | | | | | | | |
| Uusimaa | 58,411 | 56 | 2053 | 51 | 662,093 | 30 | 29,604 | 22 |
| South | 10,437 | 10 | 410 | 10 | 277,750 | 13 | 18,164 | 13 |
| West | 26,456 | 25 | 1095 | 28 | 785,408 | 35 | 48,093 | 36 |
| East | 5042 | 5 | 216 | 6 | 224,582 | 10 | 17,741 | 14 |
| North | 4770 | 4 | 212 | 5 | 266,968 | 12 | 20,455 | 15 |
| *Type of residence* | | | | | | | | |
| Urban | 85,813 | 87 | 3289 | 85 | 1,543,980 | 70 | 83,159 | 63 |
| Rural | 12,392 | 13 | 569 | 15 | 652,246 | 30 | 49,275 | 37 |
| *Unemployment days* | | | | | | | | |
| 0 | 71,467 | 68 | 2146 | 54 | 1,815,392 | 82 | 95,886 | 71 |
| 1-196 | 19,707 | 19 | 858 | 21 | 241,924 | 11 | 19,750 | 15 |
| Over 196 | 13,942 | 13 | 982 | 25 | 159,515 | 7 | 18,421 | 14 |
| *Country of Origin* | | | | | | | | |
| EU countries | 14,713 | 14 | 390 | 10 |  | | | |
| Russia/ex-Soviet Union | 43,504 | 43 | 1631 | 42 |  | | | |
| Asian countries | 18,937 | 18 | 557 | 14 |  | | | |
| Refugee-exporting countries | 12,846 | 12 | 700 | 18 |  | | | |
| Other countries | 14,142 | 14 | 634 | 16 |  | | | |
| *Length of stay* | | | | | | | | |
| 3-6 | 31,403 | 32 | 828 | 22 |  | | | |
| 7-12 | 26,784 | 28 | 908 | 25 |  | | | |
| 13-18 | 24,085 | 25 | 1123 | 31 |  | | | |
| Over 18 | 14,888 | 15 | 805 | 22 |  | | | |
